# Supplementary material for: Real-space heterogeneous reconstruction, refinement, and disentanglement of CryoEM conformational states with HetSIREN
Source: Nat Commun. 2025 Apr 22;16:3751. doi: 10.1038/s41467-025-59135-0 (PMC12015509; doi:10.1038/s41467-025-59135-0)
Supplement: Supplementary file 1 — Supplementary Information [file 41467_2025_59135_MOESM1_ESM.pdf]

Supplementary Information

Real-space heterogeneous reconstruction, refinement, and disentanglement of CryoEM conformational states with HetSIREN

David Herreros[*1], Carlos Perez Mata[1,2], Chari Noddings[3], Deli Irene[4], James Krieger[1], David A. Agard[5,6], Ming-Daw Tsai[4], Carlos Oscar Sanchez Sorzano[+1], and Jose Maria Carazo[+1]

[1]Centro Nacional de Biotecnologia-CSIC, C/ Darwin, 3, 28049, Cantoblanco, Madrid, Spain
[2]PKF Attest innCome, Orense 81, 28020, Madrid
[3]Altos Labs, Redwood City, CA, USA
[4]Institute of Biological Chemistry, Academia Sinica, Taipei 115, Taiwan
[5]Department of Biochemistry Biophysics, University of California, San Francisco, CA, USA
[6]Chan Zuckerberg Imaging Institute, Redwood City, CA, USA
[+]These authors jointly supervised this work: C.O.S. Sorzano and J.M. Carazo

* Corresponding author
E-mail: dherreros@cnb.csic.es

Supplementary Methods

Cryo-EM sample preparation and data collection for the SARS-CoV-2 samples

0.5 mg/ml of purified Beta variant Spike protein sample in 1X PBS buffer at pH 7 was diluted by 100 mM sodium citrate tribasic dihydrate at pH 5 to a final concentration of 0.25 mg/ml and a final pH value of 5.5, a condition in which the preferred orientation was minimized. A 4 μl sample was applied to Quantifoil holey carbon grids R1.2/1.3 200 mesh for 4°C, and to Quantifoil gold grids R2/2 200 mesh for 37°C, with prior incubation at the respective temperatures for 10 min. The grids had been glow discharged with negative polarity at 25 mA for 30 seconds using an EMS 100 Glow discharge apparatus. They were used within 30 min to minimize the hydrophilic recovery of the grids. After application of the sample, the grids were incubated for 10 seconds in 100% humidity at 4°C or 37°C in a Mark IV vitrobot device (Thermo Fisher Scientific) and then blotted for 3 seconds with blot force 0 before being flash frozen in liquid ethane cooled by liquid nitrogen.

For the 4°C and 37°C samples, 11,137 and 7,064 movie micrographs were automatically collected on a Bio-quantum-K3 detector (Gatan, Inc.) at a nominal magnification of 81,000x which resulted in a pixel size of 1,061 Å by using a Titan Krios microscope (Thermo Fisher Scientific) operating at 300 keV with a GIF Quantum energy filter with a slit width of 20 eV. 50 frames per movie were collected at 1 e-/ Å^2 per frame for a total dose of 50 e-/ Å^2 on the sample by using counting mode at a defocus range between -1.5 μm to -2.2 μm.

Standard image processing workflow for the SARS-CoV-2 samples

All image processing steps were performed within the Scipion software framework (1). For both samples, particles were previously pooled through standard 2D classification approaches in CryoSPARC (7) conducted by the laboratory of Prof. Ming-Daw Tsai. These particles were then directly imported into Scipion, with 662,379 and 468,911 particles for the samples at 4°C and 37°C, respectively. The selected particles were downsampled to 1.4 Å/px. These particles generated four *ab initio* models imposing C3 symmetry in CryoSPARC (7). All particles were subjected to non-uniform refinement using the best initial model low-pass filtered to 30 Å as a reference. This refinement was followed by an angular consensus protocol (8), retaining the best 615,000 and 410,000 particles for the samples at 4°C and 37°C, respectively. We then symmetry-relaxed these C3 symmetry-refined particles into C1 (9) while performing a 3D classification into 10 classes as implemented in Relion (10). We employed a 3D clustering consensus protocol to retain stable and statistically significant particles across the entire datasets to minimize the variability in class distribution over replicates of the same protocol. We inspected the particle clusters with a p-value < 0.05, and to confirm the assignment of particles to the different conformations, we generated initial models and non-uniform refined them independently. We then rejected the clusters of particles resulting in junk volumes and selected only the best clusters, corresponding to 479,908 and 309,062 particles for the samples at 4°C and 37°C, respectively. After this standard image processing workflow, we merged all clusters for each independent sample and subjected the corresponding particles to HetSIREN.

Model building and refinement of the SARS-CoV-2 samples

Firstly, we manually docked the model into the density as a rigid body, followed by real space fitting using the Fit in Map routine in UCSF Chimera (11) for the complete Spike structure, which includes chains A, B, and C. We used previously deposited structures as starting models, matching each detected conformation: PDB IDs 7WEV and 7VX1 for Beta variant in the 3 Down and 1 Up states, respectively (12). For the 2 Up state, we computationally modified the 1 Up model (7VX1) by removing one of the RBD Down chains and replacing it with a previously duplicated and individually fitted RBD Up chain. Real-space refinement was then performed in Phenix (13) with the enabled global minimization, local grid search, ADP, and rigid body options. We defined each chain's NTD, RBD, and S2 domains as independent rigid bodies, resulting in 9 rigid bodies in total. To preserve the general arrangement of the different domains within the Spike protein, the starting model was used as a reference model with restraints and secondary structure restraints. The resulting models were then manually inspected in Chimera (11) and Coot (14) to check the fit to the density. The quality of the obtained models was assessed using MolProbity (15) as implemented in Phenix (16) and the Worldwide PDB (wwPDB) OneDep System (https://deposit-pdbe.wwpdb.org/deposition). Refinement statistics are listed in Supplementary Table 3 and 4.

Characterization of the decoupling effect on conformational landscapes

The decoupling architecture introduced in HetSIREN minimizes the effects that the pose and CTF have on the organization of the different images in a latent space. Ideally, a conformational latent space that considers only the structural differences in the particle images should be learned. This way, the conformational landscape of the biomolecule under study can be properly reflected.

To better reflect these effects in the conformational latent spaces, we propose two scenarios in which the pose and CTF's downstream effects dominate.

The first test case consists of analyzing the simulated adenylate kinase dataset presented in the first section of the manuscript. During the simulation, a different CTF corruption was applied to each projection individually, trying to make the CTF as prominent as possible against the pose and the conformational variability captured in the images.

The previous images were used to train two different HetSIREN networks. The first network has a standard architecture without decoupling, while the second network includes the CTF decoupling architecture but not the pose decoupling part. Comparing the two landscapes allows one to better observe how the CTF decoupling architecture affects the latent space organization.

The results of this analysis are summarized in Supplementary Figure 8. The landscape colors represent a clustering of the different images according to their CTF information to simplify the visualization of the organization of the images based on the CTF information. Supplementary Figure 8a shows the landscape learned by the network with no decoupling architecture, leading to a significant landscape spreading to accommodate different "bands" with similar CTFs. This is a strong deviation from the gold standard landscape, which should be a straight line, as discussed in the manuscript section "Simulated adenylate kinase landscape and landscape disentanglement." In contrast, the CTF-decoupled latent space shown in Supplementary Figure 8b shows a more condensed latent space, better reflecting the ideal latent space. By combining images with the same conformation and variable CTF information, HetSIREN effectively learns

to decouple the CTF effect, minimizing its effect on the organization of the latent space and leading to a more prominent structural component.

The second test relies on analyzing clean images without CTF, which allows us to better assess the pose effect on the conformational landscape. To that end, we simulated 2000 images from two SARS-CoV-2 Spike electron density maps in one-up and three-down conformations. This simulated dataset describes a very simple conformational latent space, ideally consisting of two isolated points representing the two discrete states used to simulate the images.

Similar to the previous test, two HetSIREN networks were trained with the new image dataset: the first network has a standard architecture with no decoupling parts, while the second includes only the pose decoupling architecture to analyze its effect on the landscape. The results of this analysis are summarized in Supplementary Figure 9. Supplementary Figure 9a shows the landscapes obtained from the training dataset, colored according to clustering into four groups of the projection sphere to better visualize the pose. The non-decoupled landscape suffers from a similar effect to the CTF case, deviating from the ideal "two dots" latent space due to a strong organization induced by the pose. In contrast, the decoupled landscape presented is significantly condensed towards the ideal "two dots" representation, showing the ability of the architecture to effectively learn that images with similar conformation and different poses should be close in the latent space. A different experiment is proposed in Supplementary Figure 9b, where the previously trained networks are used to predict the landscape of a new dataset composed of the original images after applying noise to their poses. As can be seen from this result, the non-decupled network predicts a disordered landscape, placing the particles in completely different locations compared to the landscape shown in panel a), even if the images are the same. In contrast, the decupled landscape is not so much affected by the new poses, as it has learned to predict that the images represent two distinct conformations independently of their pose.

Comparison of SIREN and ReLU activation in HetSIREN

Applying different activation functions to the outputs of the layers in a neural network may induce differences in the accuracy and performance of a neural network. In this manuscript, we propose the application of sine activation functions well known in the deep learning field as SIRENs. Even though SIREN activation functions usually outperform other popular activations like ReLU, it is interesting to evaluate their effect on HetSIREN and its architecture.

To properly assess the differences between SIREN and ReLU in HetSIREN, we propose a test with the EMPIAR 10028 (2) dataset analyzed throughout the manuscript, which will be used to train two networks: the first one consists of HetSIREN with ReLU activations without adding the decoupling and the additional cost functions proposed in this work to isolate the effect of the activation function. The second network follows the same principles as the first one, changing the activation function to the SIREN activations presented in this work.

After training the two networks, two different conformations were selected from the conformational landscapes, decoding two volumes representing two distinct compositional states found in the dataset: one of the conformations loses completely its 40S subunit, while the second has a smaller loss of mass in the 40S subunit of the ribosome. The comparison of these two states is presented in Supplementary Figure 10.

As can be seen from the decoded volumes, both ReLU and SIREN perform similarly in our network architecture regarding the structural details of the structures. However, a significant difference is highlighted in the upper images arising from the change in the activation function.

While ReLU activations prevent the network from learning how to completely remove the 40S subunit of the ribosome, SIREN activations lead to a more sensible representation of this evasive state thanks to a clearer removal of the subunit.

Cost function ablation studies

As discussed in the Methods manuscript section, HetSIREN implements different cost functions directly affecting the decoded volume representation, trying to guide the network toward learning more accurate and interpretable 3D volumes from the images.

One of the effects of the proposed cost functions is to directly tackle the noise present in the images, allowing the network to focus on the signal instead of learning how to add noise to the decoded volumes. To better assess the effect of the previous denoising, we proposed an ablation test starting from the simulated adenylate kinase images already described in the manuscript. The tests first analyze the set of noise-free images, followed by a progressive addition of noise. In all these steps, two HetSIREN networks were trained: one did not include the additional denoising cost functions, unlike the second network, which is allowed to learn how to denoise the decoded volumes. It is important to highlight that the three denoising cost functions proposed in the manuscript (L1 regularization and the two versions of the total variation) are evaluated together as they complement each other to reduce the denoise while preserving the relevant details.

The results obtained from the previous analysis are summarized in Supplementary Figure 11. As explained before, the first step is analyzing the original 500 noise-free images. The projections of the decoded volumes show that both HetSIREN networks could identify the correct structure. However, it is possible to observe a non-uniform background when the denoising cost functions are not included, probably generated as a CTF effect. As expected, the network properly detected the conformational change captured by the images.

The analysis continuous with a new set of noisy images simulated to have a medium noise. When medium noise is added, it is possible to observe a more drastic effect on the two neural networks. The network without denoising adds a considerable amount of noise to the decoded volume in an attempt to match the denoise of the projection, unlike the network with denoising that manages to get a noise-free volume similar to one obtained with the baseline images. Similarly to the previous case, the detected conformational change is the expected one.

Lastly, a dataset with a high level of noise was analyzed. When no denoising is considered, the decoded volumes lack any meaningful signal and are completely dominated by noise. However, the network with denoising manages not only to detect the right signal but also to produce a noise-free decoded volume with features similar to those of the baseline dataset. This result shows the strong effect of handling the noise directly with the network, allowing it to learn accurate and meaningful structure representations even in highly noisy conditions. Moreover, the network with denoising also manages to properly detect the expected conformational change, showing the ability of the network to perform heterogeneous reconstruction with small and noisy datasets.

The next set of cost functions to be evaluated are those related to the focused reconstruction/refinement introduced in the manuscript. The main purpose of these const functions is to regularize the neural network so that it learns to refine the map while preserving the original voxel value characteristics in the reference volume given to the network. Similarly to the case before, it is required to consider these cost functions simultaneously to properly evaluate their

effect, as their combination is needed to properly represent the voxel value distribution in the original volume.

For this test, we trained two neural networks using the EMPIAR 10028 dataset to simplify comparing the results with those presented in the manuscript. The only difference between the two networks trained is the consideration of the cost functions related to the focused reconstruction process. The results from this test are summarized in Supplementary Figure 12. As can be seen from the figure, when the cost functions are not considered, the network introduces a strong artifact in the decoded volume. This artifact arises from the freedom the network has to place any possible voxel value in a given position in the grid, completely breaking the relation of the decoded values with the original distribution of voxel values in the reference volume. In contrast, the regularized network effectively learns to refine the region of interest while considering that the range of values of the decoded region should be as similar as possible to the reference volume. Thus, the regularized network does not present the artifact previously described, improving the representation and interpretability of the decoded volume.

Apart from the denoising and focused reconstruction-related losses previously evaluated, HetSIREN includes an internal sharpening arising as a post-processing effect from the way the decoded volumes are constructed, which is added to the enhancement effects of the additional cost functions. However, this internal sharpening does not prevent further post-processing of the decoded volume to further enhance the structural features in the volume, which is an essential step to properly understand and interpret a given biomolecular structure.

To better reflect the previous idea, we propose a comparison of HetSIREN when the internal sharpening and a further post-processed version of the decoded volume with DeepEMhancer (17) and EMReady (18). To that end, we compared one of the HetSIREN volumes decoded for the EMPIAR-10028 dataset previously discussed in the manuscript. The comparison is presented in Supplementary Figure 13. We propose as a baseline of the comparison the CryoSPARC volume reconstructed from this dataset. The comparison shows how the internal sharpening of HetSIREN significantly improved the structural features in the volume, which are similar to the ones present in the CryoSPARC volume post-processed with DeepEMhancer. In addition, the sharpening post-processing of the HetSIREN volume enhances even further the structural features compared to its non-sharpened version and the sharpened CryoSPARC volume.

References

[1] J.M. de la Rosa-Trevín, A. Quintana, L. del Cano, A. Zaldívar, I. Foche, J. Gutiérrez, J. Gómez-Blanco, J. Burguet-Castell, J. Cuenca-Alba, V. Abrishami, J. Vargas, J. Otón, G. Sharov, J.L. Vilas, J. Navas, P. Conesa, M. Kazemi, R. Marabini, C.O.S. Sorzano, and J.M. Carazo. Scipion: A software framework toward integration, reproducibility and validation in 3D electron microscopy. *Journal of Structural Biology*, 195(1):93–99, 2016.

[2] W. Wong, X. Bai, A. Brown, I.S. Fernandez, E. Hanssen, M. Condron, Y.H. Tan, J. Baum, and S.H.W. Scheres. CryoEM structure of the *Plasmodium falciparum* 80s ribosome bound to the anti-protozoan drug emetine. *eLife*, 3:e03080, 2014.

[3] L. McInnes, J. Healy, N Saul, and L. Großberger. Umap: Uniform manifold approximation and projection. *Journal of Open Source Software*, 3(29):861, 2018.

[4] CM Noddings, J.L. Johnson, and D. Agard CryoEM reveals how Hsp90 and FKBP immunophilins co-regulate the glucocorticoid receptor. *Nature Structural & Molecular Biology*, 30:1867–1877, 2023.

[5] I. Jolliffe and J. Cadima. Principal component analysis: A review and recent developments. *Philosophical Transactions of the Royal Society A: Mathematical, Physical and Engineering Sciences*, 374:20150202, 2016.

[6] K. Jamali, L. Käll, R. Zhang, A. Brown, D. Kimanius, and S. H. W. Scheres. Automated model building and protein identification in cryo-EM maps. *Nature*, 628(8007):450–457, Apr 2024.

[7] A. Punjani, J.L. Rubinstein, D.J. Fleet, and M.A. Brubaker. CryoSPARC: algorithms for rapid unsupervised CryoEM structure determination. *Nature Methods*, 14:290–296, 2017.

[8] J.M. de la Rosa-Trevín, J. Otón, R. Marabini, A. Zaldívar, J. Vargas, J.M. Carazo, and C.O.S. Sorzano. Xmipp 3.0: An improved software suite for image processing in electron microscopy. *Journal of Structural Biology*, 184:321–328, 2013.

[9] V. Abrishami, S. L. Ilca, J. Gomez-Blanco, I. Rissanen, J. M. de la Rosa-Trevín, V. S. Reddy, J. -M. Carazo, and J. T. Huiskonen. Localized reconstruction in Scipion expedites the analysis of symmetry mismatches in cryo-EM data. *Prog. Biophys. Mol. Biol.*, 160:43–52, 2021.

[10] D. Kimanius, L. Dong, G. Sharov, T. Nakane, and S.H.W. Scheres. New tools for automated CryoEM single-particle analysis in RELION-4.0. *Biochemical Journal*, 478:4169–4185, 2021.

[11] E.F. Pettersen, T.D. Goddard, C.C. Huang, E.C. Meng, G.S. Couch, T.I. Croll, J.H. Morris, and T.E. Ferrin. Ucsf chimerax: Structure visualization for researchers, educators, and developers. *Protein Science*, 30(1):70–82, 2021.

[12] Y. Wang, C. Xu, Y. Wang, Q. Hong, C. Zhang, Z. Li, S. Xu, Q. Zuo, C. Liu, Z. Huang, and Y. Cong. Conformational dynamics of the Beta and Kappa SARS-CoV-2 spike proteins and their complexes with ACE2 receptor revealed by cryo-EM. *Nat. Commun.*, 12(1):7345, 2021.

[13] D. Liebschner, P.V. Afonine, M.L. Baker, G. Bunkóczi, V.B. Chen, T.I. Croll, B. Hintze, L.W. Hung, S. Jain, A.J. McCoy, N.W. Moriarty, R.D. Oeffner, B.K. Poon, M.G. Prisant, R.J. Read, J.S. Richardson, D.C. Richardson, M.D. Sammito, O.V. Sobolev, D.H. Stockwell, T.C. Terwilliger, A.G. Urzhumtsev, L.L. Videau, C.J. Williams, and P.D. Adams. Macromolecular structure determination using X-rays, neutrons and electrons: recent developments in Phenix *Acta Cryst. D*, 75:861–877, 2019.

[14] P. Emsley and K. Cowtan, Coot: model-building tools for molecular graphics. *Acta Crystallogr. D Biol. Crystallogr.*, vol. 60, no. Pt 12 Pt 1, pp. 2126–2132, 2004.

[15] V. B. Chen, W. B. Arendall III, J. J. Headd, D. A. Keedy, R. M. Immormino, G. J. Kapral, L. W. Murray, J. S. Richardson, and D. C. Richardson. MolProbity: all-atom structure validation for macromolecular crystallography. *Acta Crystallogr. D Biol. Crystallogr.*, 66(Pt 1):12–21, 2010.

[16] C. J. Williams, J. J. Headd, N. W. Moriarty, M. G. Prisant, L. L. Videau, L. N. Deis, V. Verma, D. A. Keedy, B. J. Hintze, V. B. Chen, S. Jain, S. M. Lewis, W. B. Arendall III, J. Snoeyink, P. D. Adams, S. C. Lovell, J. S. Richardson, and D. C. Richardson. MolProbity: More and better reference data for improved all-atom structure validation. *Protein Sci.*, 27(1):293–315, 2018.

[17] R. Sanchez-Garcia, J. Gomez-Blanco, A. Cuervo, J.M. Carazo, C.O.S Sorzano, and J. Vargas. DeepEMhancer: a deep learning solution for cryo-EM volume post-processing. *Communications Biology*, 4:874, 2021.

[18] He J., Li T., and Huang S.Y. Improvement of cryo-EM maps by simultaneous local and non-local deep learning. *Nat. Commun.*, 14:3217, 2023.

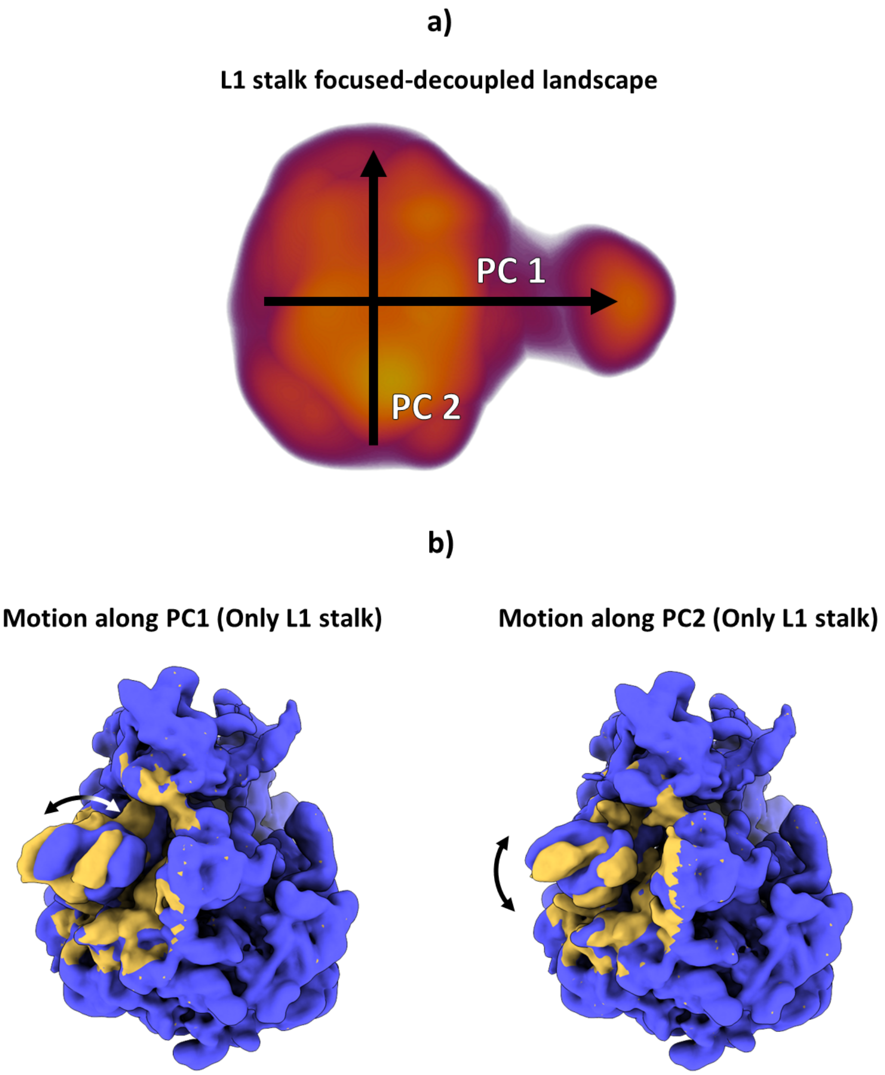

Supplementary Figure 1: Example of the L1 stalked-focused landscape estimated with HetSIREN. The landscape was estimated with the pose and CTF decoupling architecture by providing a spherical mask to the network enclosing the L1 stalk. This way, HetSIREN will only consider the L1 stalk region when determining the motions and conformational changes captured in the experimental particle images. Panel a) shows the UMAP (3) representation of the conformational latent space, including the approximate principal direction according to PCA (5). Panel b) shows the main L1 stalk motions detected by HetSIREN when sampling along the conformational latent space's first and second principal components. The motion detected shows a strong lateral and vertical displacement of the L1 stalk, which is much more easily identified here than when considering the whole particle, thanks to the ability to focus the landscape in this specific region.

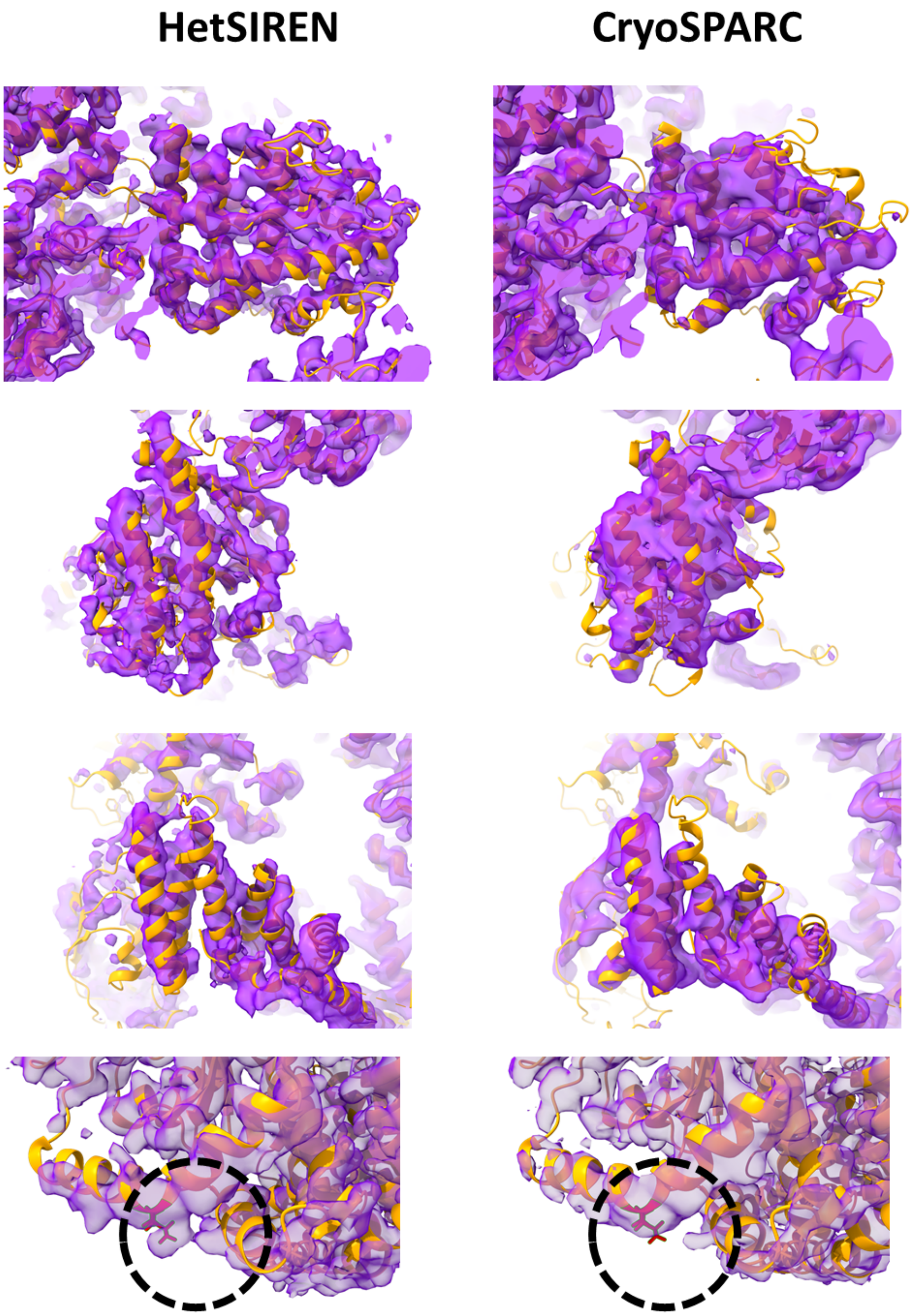

Supplementary Figure 2: Detailed comparison of HetSIREN and the deposited map from (4). The different panels present several zoom regions of the two volumes to better compare the resolution changes between HetSIREN and the deposited map. In addition, we highlight in the last row how HetSIREN has the ability to detect small structural details like side chains in the decoded volumes.

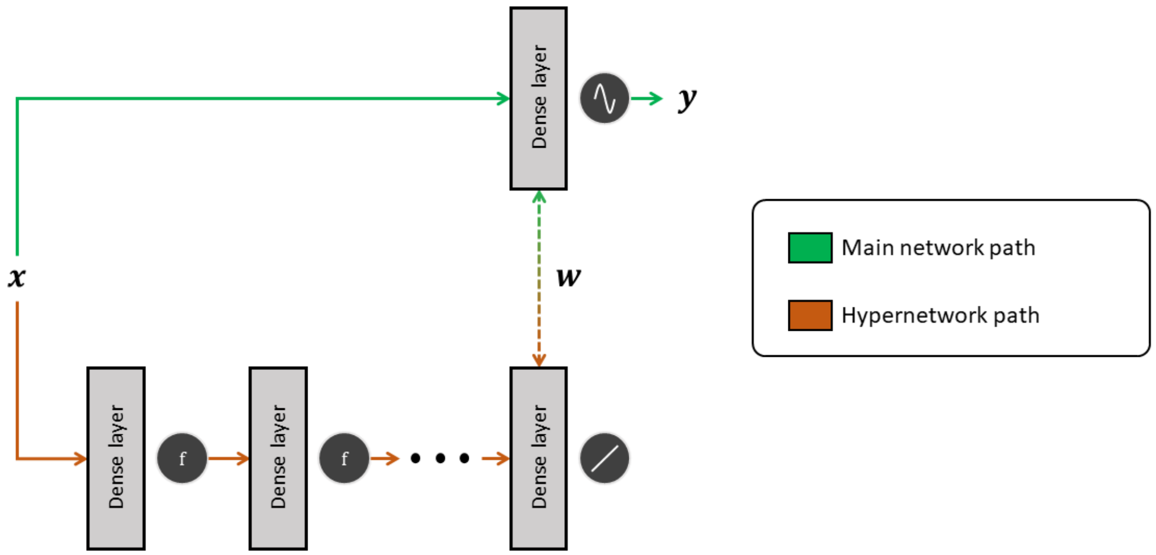

Supplementary Figure 3: Scheme of a meta-sinusoidal layer as implemented in the HetSIREN volume decoder network. The proposed architecture relies on a fully connected network with several layers (hypernetwork) whose weights will be updated during the backpropagation phase. The weights of the last layer in the fully connected network are then shared with the dense layer with the sine activation so that it can decode the appropriate outputs.

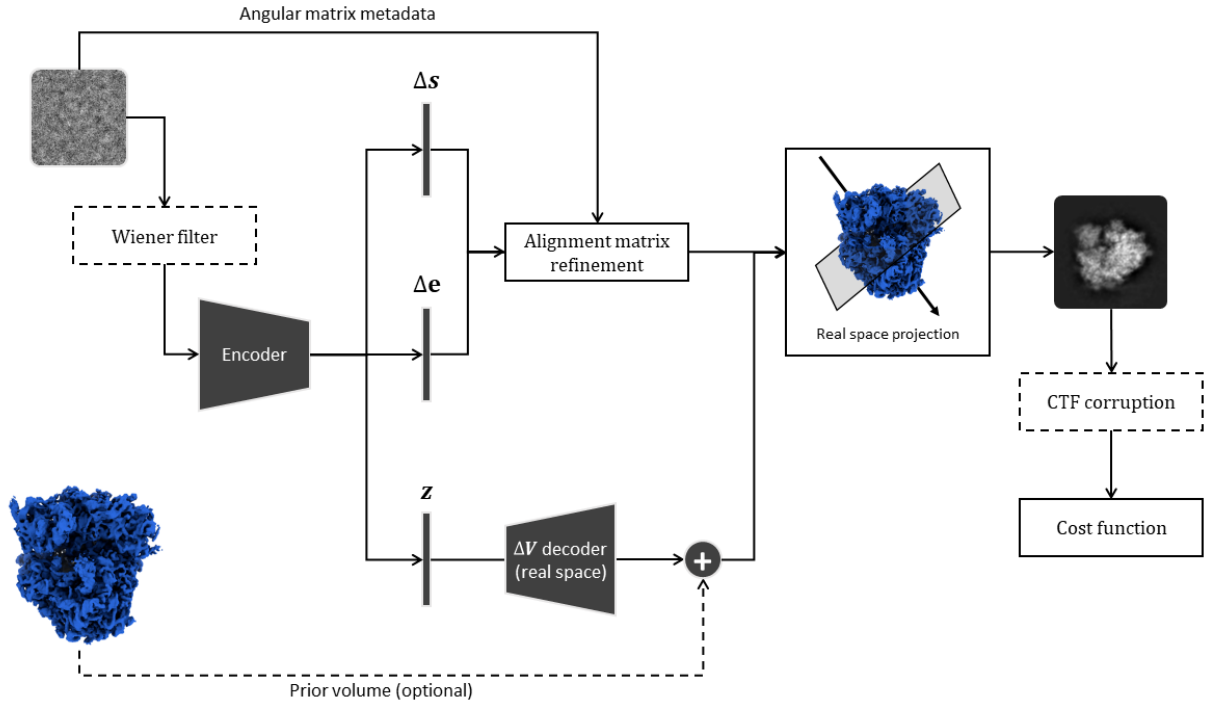

Supplementary Figure 4: Scheme of the HetSIREN network architecture and training strategy. In the scheme, the encoder has a dynamic architecture based on the user inputs (available choices include fully connected and convolutional architectures). The $\Delta\mathbf{V}$ decoder directly produces a full 3D volume in real space from the encoded latent space vectors $\mathbf{z}$. Depending on the availability of the prior volume, the decoded $\Delta\mathbf{V}$ could translate into a full reconstruction (without the prior volume) or a refinement. In addition to the conformational latent space $\mathbf{z}$, two additional bottleneck layers are estimated: a $\Delta\boldsymbol{s}$ layer to refine the in-plane shift of the particle and a $\Delta\boldsymbol{e}$ layer to refine the particle projection angle. The previous two vectors are combined to refine the estimated alignment matrices associated with the experimental image. Regarding the CTF, three possible scenarios are considered: particles have been previously corrected (no CTF considered inside the network), particles are CTF corrected before being fed to the encoder (Wiener filter box), or theoretical projections are CTF corrupted (CTF corruption box).

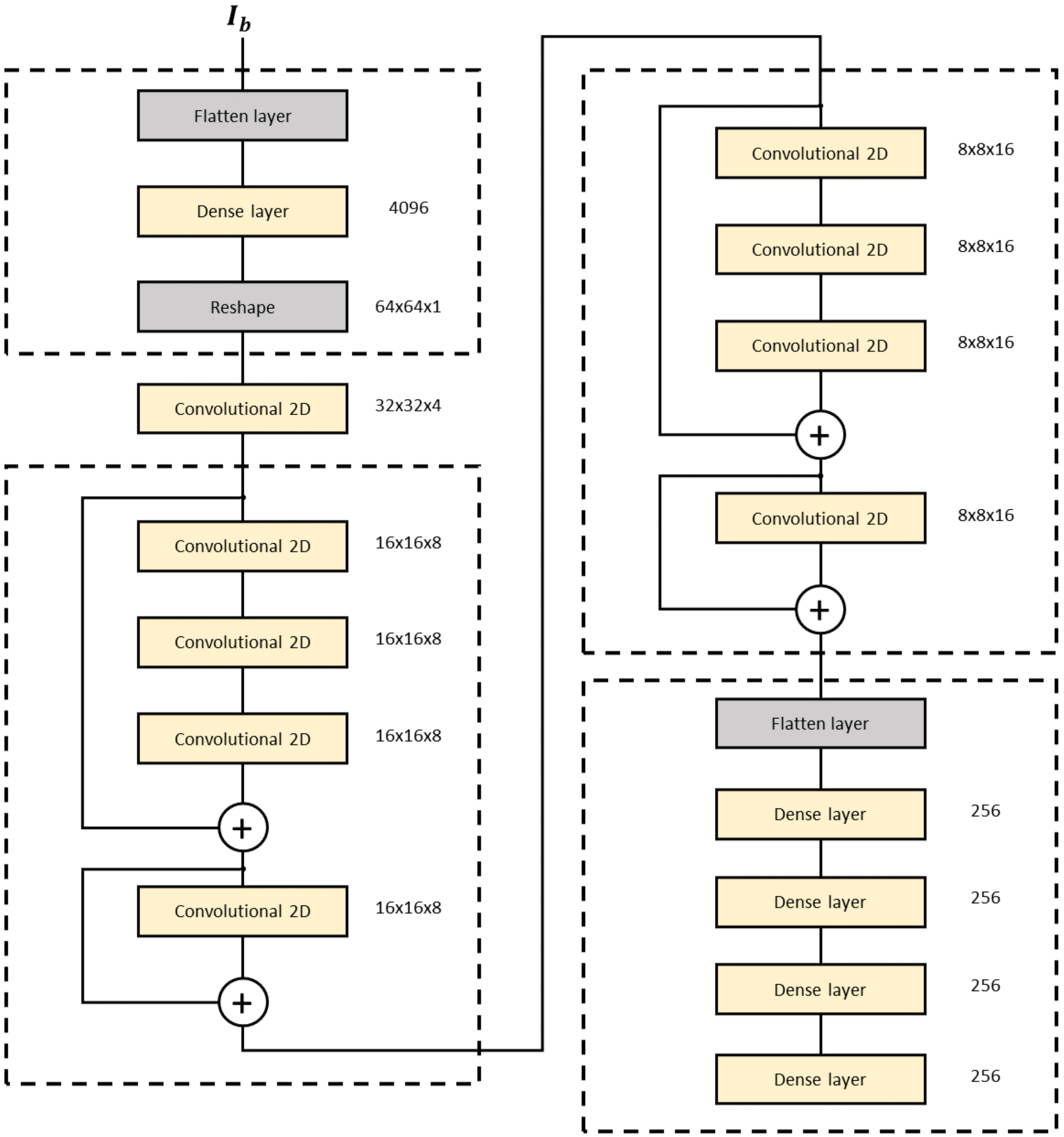

Supplementary Figure 5: Example of the default encoder architecture implemented in HetSIREN. The encoder relies on a resizing network followed by convolutional blocks with residual skips. The output images from the residual blocks are then passed to a fully connected block whose output is posteriorly converted into the three bottlenecks defined in HetSIREN.

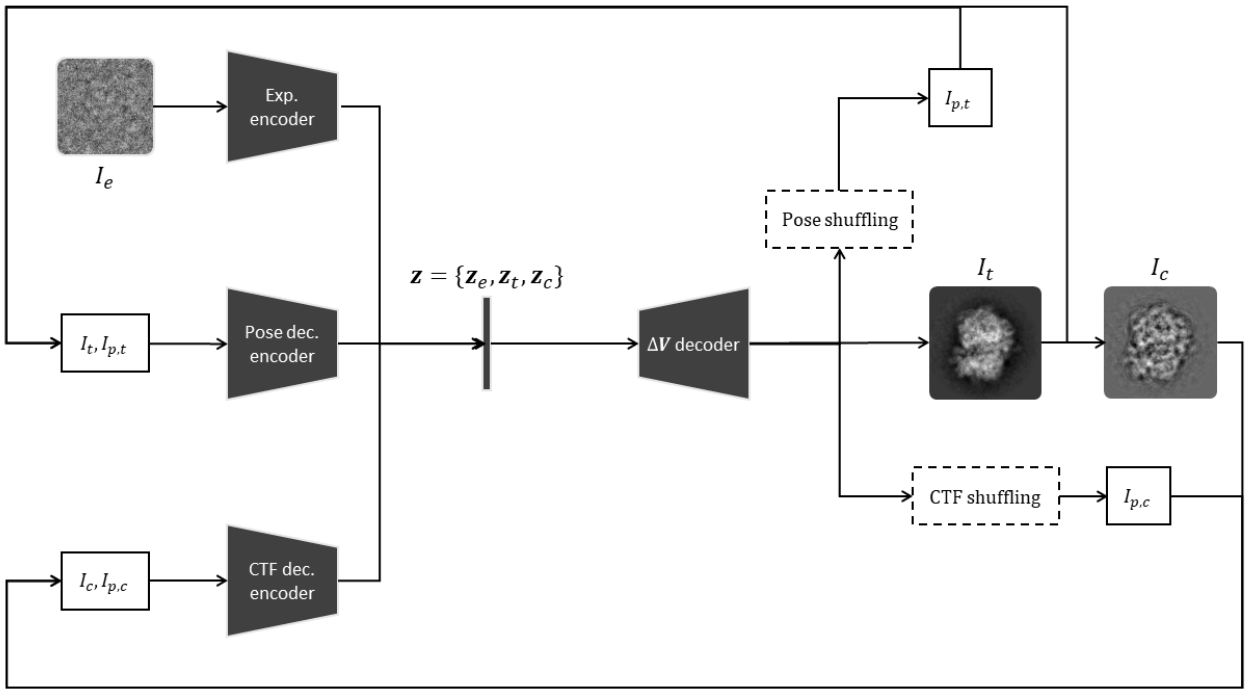

Supplementary Figure 6: HetSIREN poses and CTF decoupling architecture. The decoupling process starts with a batch of experimental images forwarded through the experimental encoder and the decoder to generate a batch of clean, and CTF corrupted projections I_t and I_c. In addition, the original poses and CTFs are shuffled to generate a new set of clean projections with the same conformation but variable pose and CTF $I_{p,t}$ and $I_{p,c}$. Once all the projections have been generated, the images I_t and $I_{p,t}$ are forwarded through the pose decoupling decoder. Similarly, the images I_c and $I_{p,c}$ are fed to the CTF decoupling decoder. In this way, it is possible to generate several sets of latent space vectors representing the same conformational state but with variable pose and CTF, which can be used to decouple the pose and CTF effects from the latent space as expressed in Equations **??** and **??**.

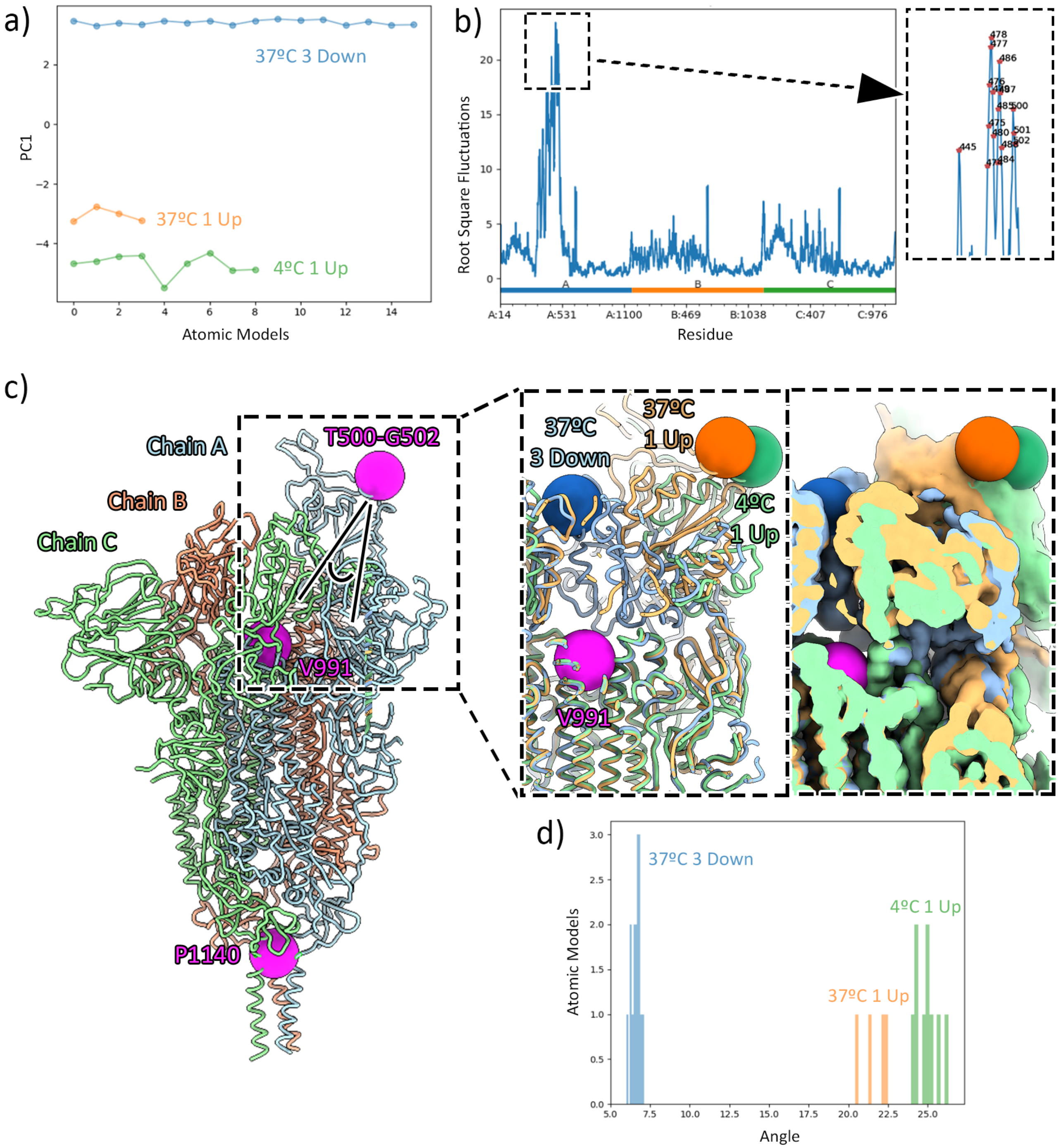

Supplementary Figure 7: Structural analyses of the SARS-CoV-2 Spike protein. Panel a) shows the PCA (5) for the atomic structures ensemble encompassing 16 models of 3 Down conformation at 37°C (blue), 4 models of 1 Up conformation at 37°C (orange), and 9 models of 1 Up conformation at 4°C (green). The Root Mean Square Fluctuations derived from PCA (5) for individual residues are shown in panel b). The inset shows a zoomed area of the residues exhibiting the highest mobility. Panel c) shows a representative atomic model for the 1 Up conformation (chain A in light blue, chain B in light red, and chain C in light green). The three magenta spheres represent the centroids used for the analyses of angle measurements (Thr500-Gly502 at the RBD and Val991 and Pro1140 at the top and bottom of the S2 domain, respectively). Angle is indicated in the dashed box. Insets show a detail of the differences between the three analyzed conformations (3 Down at 37°C in light blue, 1 Up at 37°C in light orange, and 1 Up at 4°C in light green) at atomic models (left) and cryoEM reconstructions (right) levels. Analyses of angle measurements are shown in panel d), matching the color code of inset c). Source data are provided as a Source Data file.

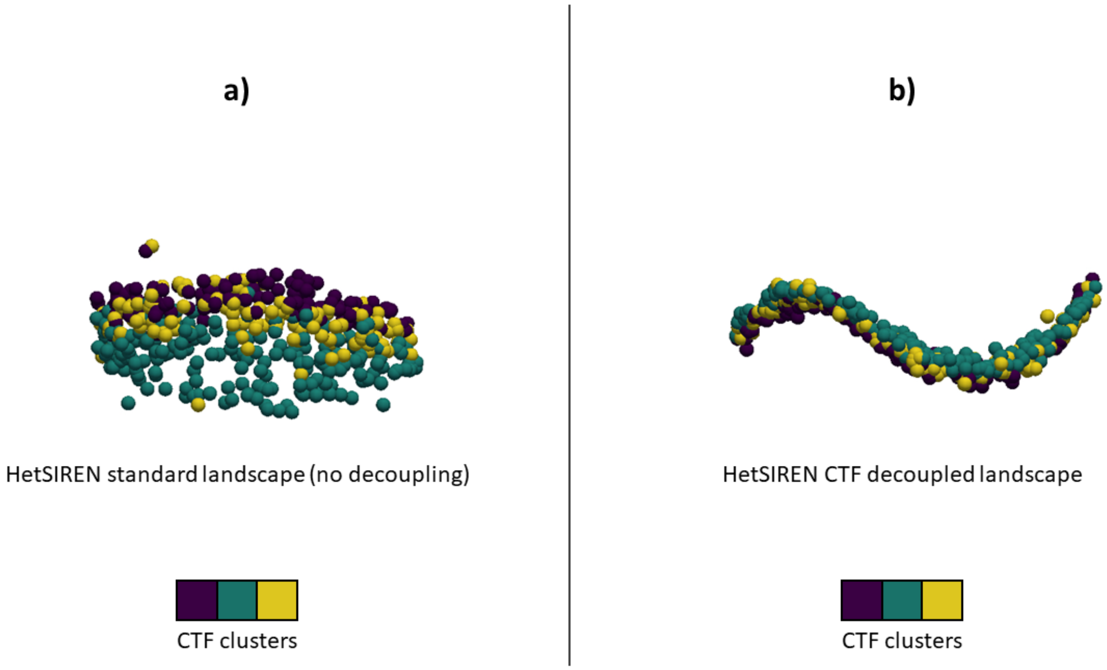

Supplementary Figure 8: Assessment of the CTF decoupling architecture on the latent space learned by HetSIREN. Panels a) and b) show two latent spaces obtained by training two different networks with images with variable CTF corruption. Panel a) shows the landscape encoded by the network with no decoupling architecture. Panel b) shows the landscape encoded by the network, including only the CTF decoupling part. The colors used to represent the landscapes correspond to a clustering of the CTF of the images into three different groups to simplify the visualization of this information. The comparison of the two panels shows how the decoupling effect effectively condenses the latent space, reducing the spreading induced by the strong organization of the latent space according to the CTF of the images.

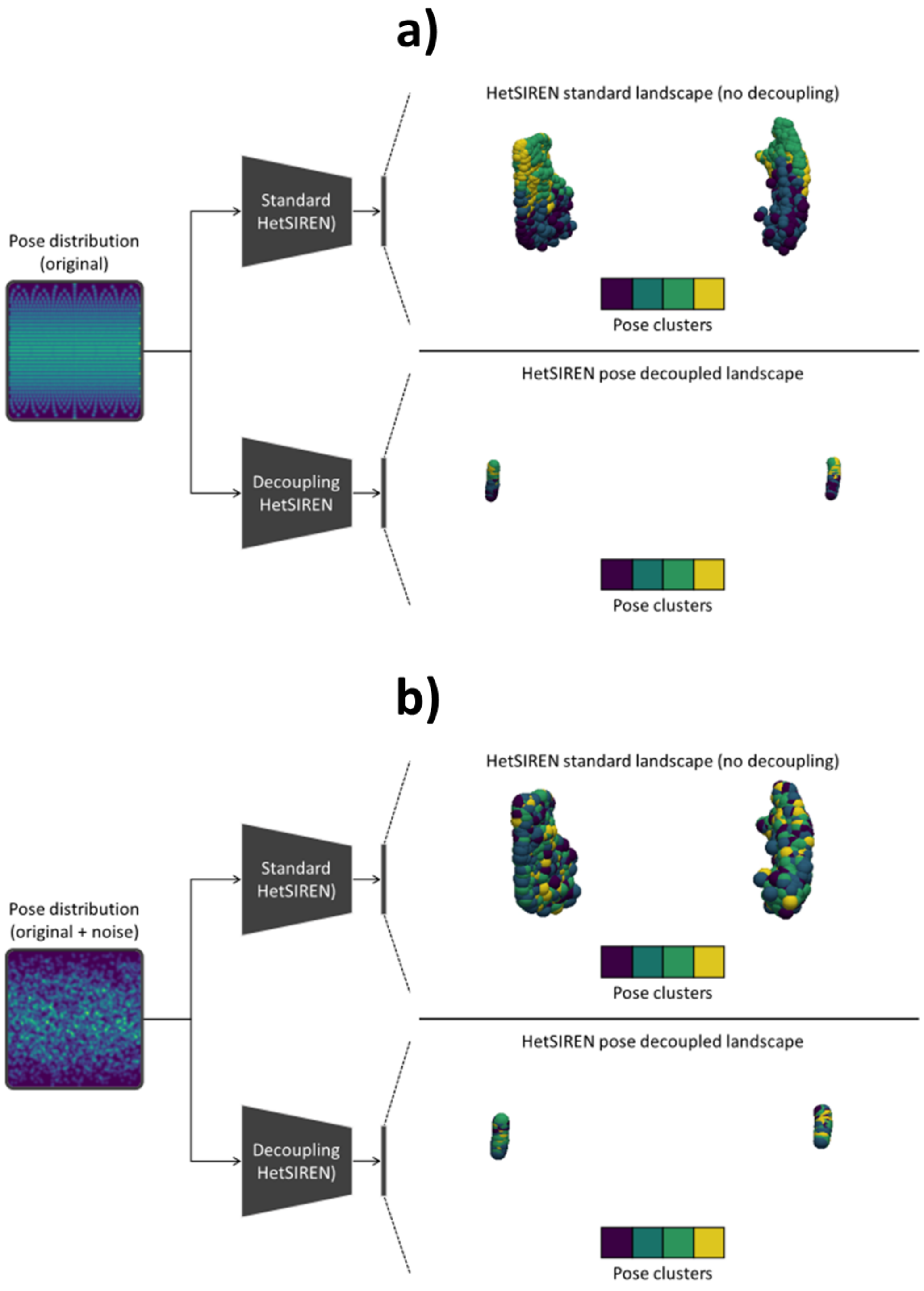

Supplementary Figure 9: Assessment of the pose decoupling architecture on the latent space learned by HetSIREN. The panels show the latent spaces obtained by training two different networks with images with variable poses and no CTF corruption. Panel a) shows the landscapes obtained with the training dataset with a uniform pose distribution. Panel b) shows the landscapes obtained after predicting from the training dataset after adding noise to the original poses. The colors used to represent the landscapes correspond to a clustering of the pose of the images into four different groups to simplify the visualization of this information. The comparison of the two panels shows how the decoupling effect effectively condenses the latent space, reducing the spreading induced by the strong organization of the latent space according to the pose of the images.

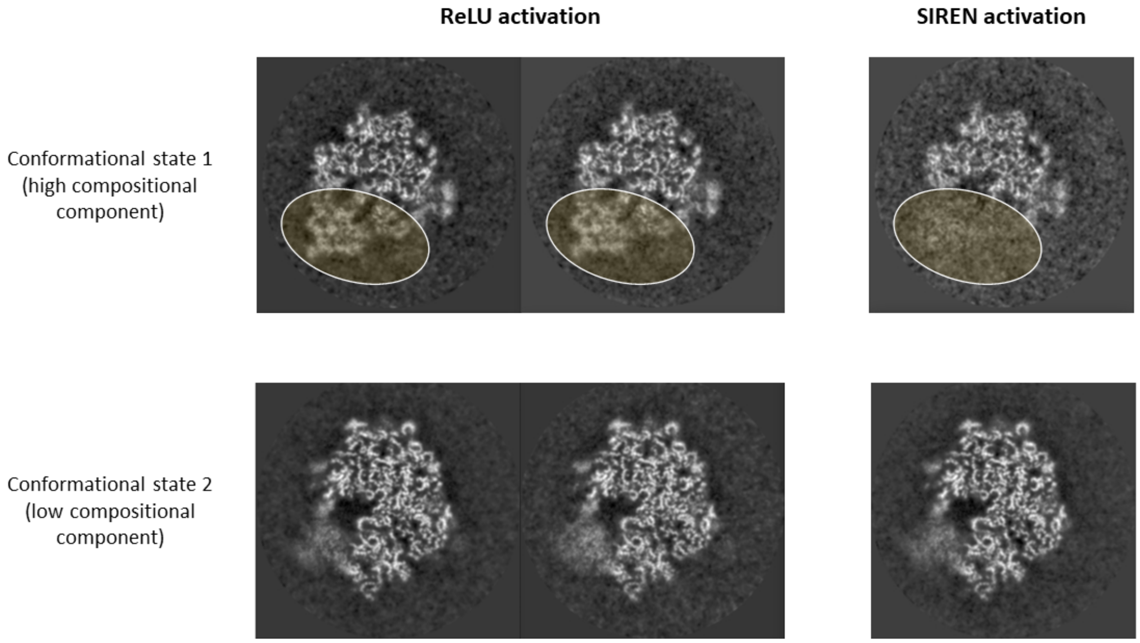

Supplementary Figure 10: Comparison of the decoded accuracy of HetSIREN when trained using two different activation functions in the decoded: ReLU and SIREN. The comparison shows that both activations have a similar performance in representing the structural details in a given state, although SIREN gives more freedom to the network to represent strong compositional variations, as highlighted in the upper images.

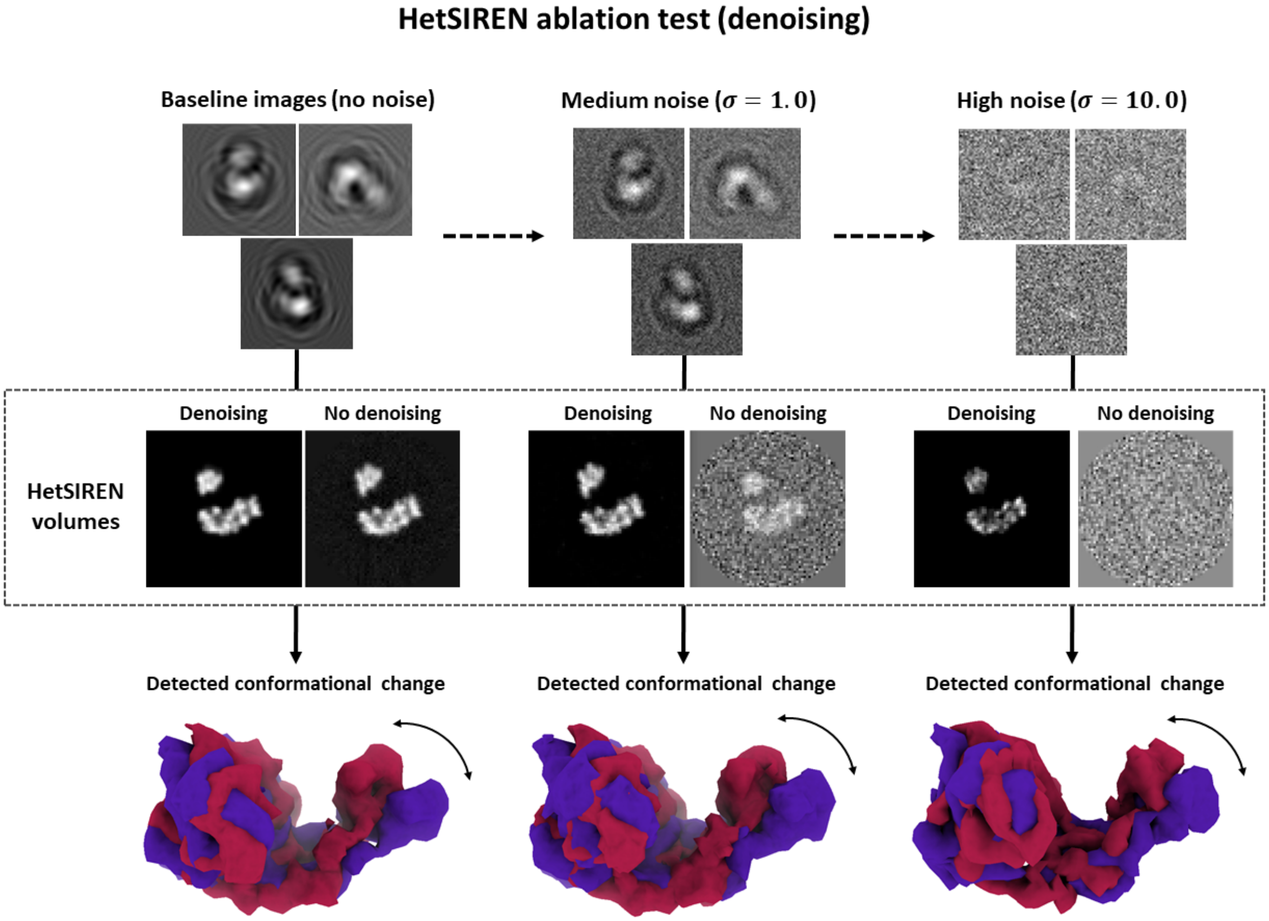

Supplementary Figure 11: Ablation test to analyze the performance of the denoising cost functions implemented during the training phase of HetSIREN. The test evaluates the denoising capabilities of the network under different noise conditions: a set of ideal images, images with medium noise ($\sigma = 1$), and high noise ($\sigma = 10$). In all cases, two different networks were trained, whose only difference is the presence of the denoising cost functions in one of them. The 3D volumes shown are decoded with the denoising network in all cases.

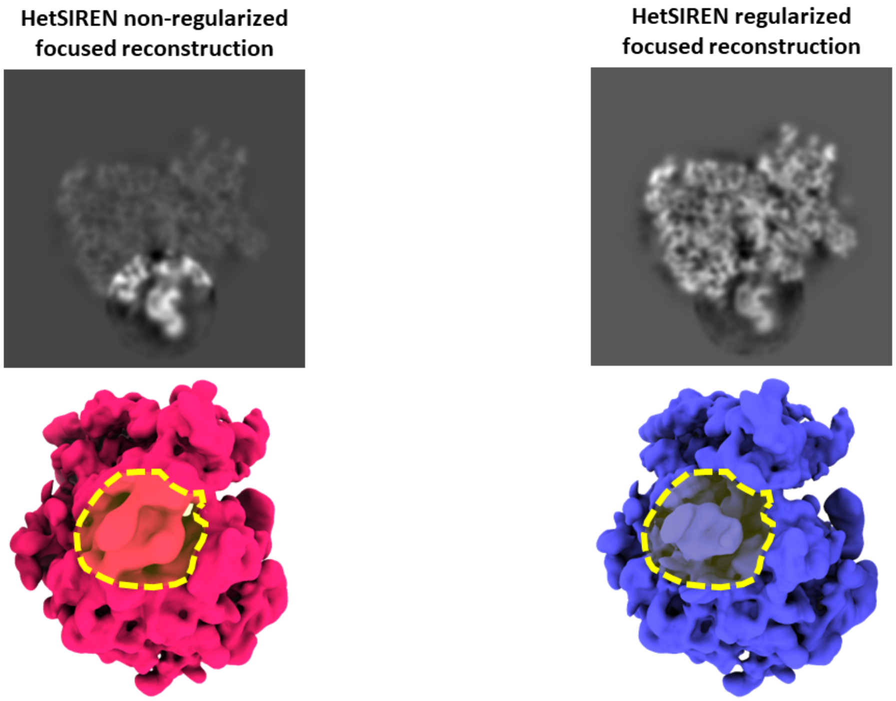

Supplementary Figure 12: Evaluation of the focused reconstruction-related cost functions implemented in HetSIREN. The test evaluates the effect of adding the cost functions responsible for ensuring that the values in chimera volume follow a similar distribution. When this regularization is not applied, the decoded volume shows a clear artifact arising from a strong difference in the value distribution of the refined region and the rest of the volume. In contrast, the regularized network properly minimizes the previous artifact, yielding a more consistent volume.

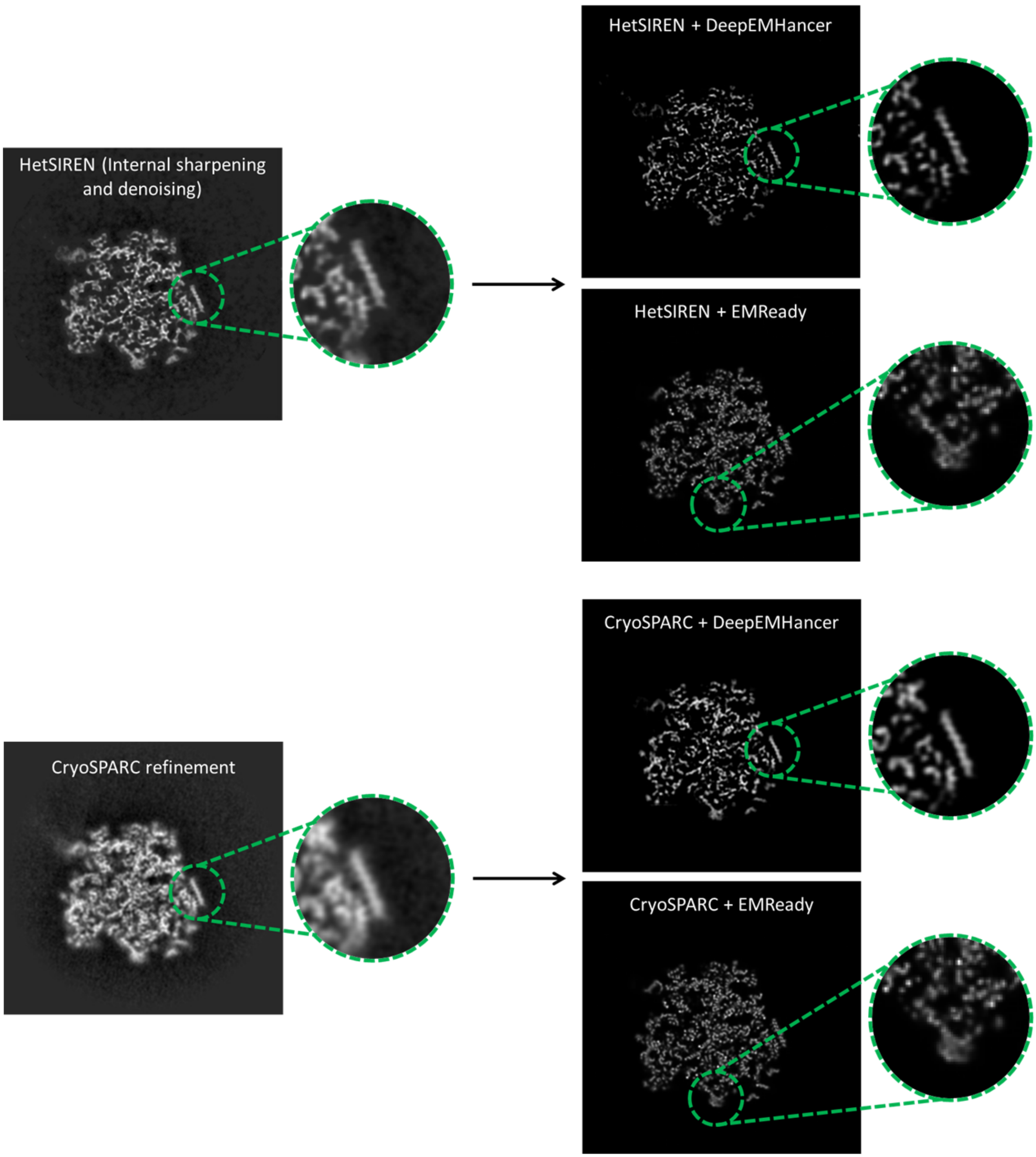

Supplementary Figure 13: Comparison of HetSIREN and CryoSPARC reconstruction for the EMPIAR 10028 (2) dataset. The comparison shows first the original volumes obtained by both approaches. In the case of HetSIREN, the decoded volume includes the internal sharpening applied during the decoding step, as described in the manuscript. In addition, the figure shows the previous two volumes further post-processed by DeepEmhancer (17) and EMReady (18) to further enhance their structural features. This comparison reveals that the internal sharpening implemented in HetSIREN does not prevent further modification of the decoded volume, yielding a new representation with significantly enhanced structural features compared to both the CryoSPARC maps and its sharpened representation.

Performance metrics for HetSIREN				
Image size	Batch size	Epochs	GPU memory (GB)	Time 10^5 particles (hours)
128	16	50 (standard)	2.42	2.75
300	8	50 (standard)	17.3	14.2
300	8	50 (disentanglement)	17.3	14.5

Supplementary Table 1: Execution times and GPU memory consumption for HetSIREN. Metrics are referred to in the training phase.

**Automatically modeled residues (ModelAngelo)**	
	**RBD (residues 304-591)**
**CryoSPARC Map**<br>**Modelled Residues**<br>**% Total Residues**	 310<br>35.9%
**HetSIREN map 4**	365<br>42.2%
**HetSIREN map 9**	342<br>39.6%
**HetSIREN map 13**	401<br>46.4%
**HetSIREN map 14**	326<br>37.7%
**HetSIREN map 15**	346<br>40.0%

Supplementary Table 2: Comparison of automatically modeled residues performed by ModelAngelo.

**Refinement statistics for SARS-CoV-2 Spike protein at 4°C**										
**Refinement**	**Map 1**	**Map 2**	**Map 3**	**Map 4**	**Map 5**	**Map 6**	**Map 7**	**Map 8**	**Map 9**	**Map 10**
Mask correlation coefficient	0.69	0.69	0.70	0.71	0.64	0.69	0.70	0.72	0.72	0.69
Model composition										
Non-hydrogen atoms	25,362	25,362	25,362	25,362	25,362	25,362	25,362	25,362	25,362	25,362
Protein residues	3,237	3,237	3,237	3,237	3,237	3,237	3,237	3,237	3,237	3,237
ADP (B-factors)										
min	56.88	65.32	54.68	67.54	47.65	52.98	59.54	50.81	63.09	62.66
max	428.31	325.45	462.43	318.58	363.74	620.24	539.35	297.54	358.72	455.62
mean	149.62	132.17	156.76	138.32	155.21	157.98	161.59	136.61	131.11	156.43
R.m.s deviations										
Bond lengths	0.007	0.007	0.007	0.007	0.007	0.007	0.007	0.007	0.009	0.008
Bond angles	1.428	1.490	1.442	1.500	1.367	1.384	1.446	1.484	1.597	1.455
Validation										
Molprobity score	1.65	1.77	1.62	1.81	1.53	1.60	1.58	1.64	1.79	1.69
Clashscore	5.91	6.35	5.95	6.31	4.97	5.77	5.55	6.49	7.64	6.99
Rotamer outliers (%)	1.17	0.74	1.06	0.99	0.78	0.67	0.88	0.99	1.24	0.60
Ramachandran plot										
Favoured (%)	95.97	93.05	95.94	95.69	96.03	95.91	96.00	95.91	95.72	95.66
Allowed (%)	3.97	6.95	4.00	4.25	3.91	4.03	3.94	4.03	4.22	4.28
Outlier (%)	0.06	0.00	0.06	0.06	0.06	0.06	0.06	0.06	0.06	0.06

**Refinement statistics for SARS-CoV-2 Spike protein at 4°C**										
**Refinement**	**Map 11**	**Map 12**	**Map 13**	**Map 14**	**Map 15**	**Map 16**	**Map 17**	**Map 18**	**Map 19**	**Map 20**
Mask correlation coefficient	0.68	0.70	0.71	0.72	0.70	0.67	0.69	0.69	0.69	0.71
Model composition										
Non-hydrogen atoms	25,362	25,362	25,362	25,362	25,362	25,362	25,362	25,362	25,362	25,362
Protein residues	3,237	3,237	3,237	3,237	3,237	3,237	3,237	3,237	3,237	3,237
ADP (B-factors)										
min	54.78	55.29	55.46	58.05	51.09	54.05	55.09	58.39	49.91	51.77
max	579.72	549.59	440.90	259.33	375.42	470.42	454.68	401.38	460.43	657.55
mean	156.63	171.29	150.96	127.42	140.63	155.53	153.43	144.97	159.05	153.25
R.m.s deviations										
Bond lengths	0.008	0.007	0.008	0.008	0.008	0.007	0.007	0.007	0.007	0.007
Bond angles	1.478	1.441	1.608	1.532	1.473	1.402	1.465	1.430	1.433	1.515
Validation										
Molprobity score	1.72	1.58	1.65	1.67	1.61	1.58	1.61	1.69	1.58	1.66
Clashscore	6.17	5.47	6.31	6.73	5.89	5.63	6.05	6.93	5.29	6.47
Rotamer outliers (%)	1.41	0.78	0.81	0.92	0.99	0.67	0.81	1.09	0.78	0.81
Ramachandran plot										
Favoured (%)	96.03	95.91	95.66	95.66	95.78	95.97	95.97	95.91	95.72	95.69
Allowed (%)	3.91	4.03	4.25	4.28	4.15	3.97	3.97	4.00	4.22	4.25
Outlier (%)	0.06	0.06	0.09	0.06	0.06	0.06	0.06	0.09	0.06	0.06

Supplementary Table 3: Refinement statistics for SARS-CoV-2 Spike protein at 4°C.

**Refinement statistics for SARS-CoV-2 Spike protein at 37°C**										
**Refinement**	**Map 1**	**Map 2**	**Map 3**	**Map 4**	**Map 5**	**Map 6**	**Map 7**	**Map 8**	**Map 9**	**Map 10**
Mask correlation coefficient	0.68	0.69	0.74	0.77	0.73	0.75	0.76	0.74	0.69	0.73
Model composition										
Non-hydrogen atoms	25,362	25,362	25,482	25,482	25,482	25,482	25,482	25,482	25,362	25,482
Protein residues	3,237	3,237	3,255	3,255	3,255	3,255	3,255	3,255	3,237	3,255
ADP (B-factors)										
min	66.33	69.60	73.86	84.37	69.29	71.30	68.22		73.47	63.30
max	469.35	418.14	418.14	331.04	299.72	277.98	319.64	261.50	412.59	336.72
mean	151.83	149.02	197.15	128.96	134.03	124.63	127.39	124.20	151.80	135.49
R.m.s deviations										
Bond lengths	0.008	0.008	0.008	0.009	0.009	0.008	0.009	0.009	0.007	0.008
Bond angles	1.603	1.612	1.717	1.840	1.829	1.745	1.777	1.797	1.566	1.738
Validation										
Molprobity score	1.69	1.65	1.61	1.80	1.62	1.66	1.59	1.63	1.68	1.63
Clashscore	6.63	6.21	6.06	7.53	6.99	6.26	6.36	6.49	6.69	5.96
Rotamer outliers (%)	1.02	0.88	1.16	1.72	0.88	1.33	1.05	1.19	0.92	1.33
Ramachandran plot										
Favoured (%)	95.44	95.50	96.49	96.71	96.49	96.58	96.52	96.61	95.60	96.71
Allowed (%)	4.47	4.40	3.42	3.20	3.42	3.32	3.39	3.29	4.31	3.20
Outlier (%)	0.09	0.09	0.09	0.09	0.09	0.09	0.09	0.09	0.09	0.09

**Refinement statistics for SARS-CoV-2 Spike protein at 37°C**										
**Refinement**	**Map 11**	**Map 12**	**Map 13**	**Map 14**	**Map 15**	**Map 16**	**Map 17**	**Map 18**	**Map 19**	**Map 20**
Mask correlation coefficient	0.67	0.74	0.75	0.76	0.74	0.74	0.74	0.74	0.75	0.74
Model composition										
Non-hydrogen atoms	25,362	25,482	25,482	25,482	25,482	25,482	25,482	25,482	25,482	25,482
Protein residues	3,237	3,255	3,255	3,255	3,255	3,255	3,255	3,255	3,255	3,255
ADP (B-factors)										
min	67.35	73.33	75.37	80.29	70.45	66.29	71.37	86.52	76.29	78.77
max	450.55	359.03	350.66	288.60	290.02	330.95	288.01	405.81	421.83	304.79
mean	152.38	130.67	127.95	128.29	127.89	129.61	125.55	131.54	262.12	125.21
R.m.s deviations										
Bond lengths	0.007	0.009	0.008	0.008	0.008	0.008	0.008	0.008	0.008	0.008
Bond angles	1.549	1.793	1.751	1.739	1.740	1.790	1.812	1.720	1.799	1.746
Validation										
Molprobity score	1.70	1.66	1.60	1.58	1.65	1.59	1.69	1.59	1.61	1.57
Clashscore	5.69	6.36	6.57	6.16	6.32	6.16	6.87	5.84	6.45	5.44
Rotamer outliers (%)	0.85	1.37	1.09	1.09	1.30	1.12	1.30	1.19	1.19	1.23
Ramachandran plot										
Favoured (%)	95.56	96.74	96.64	96.68	96.64	96.64	96.55	96.64	96.77	96.71
Allowed (%)	4.34	3.17	3.26	3.23	3.26	3.26	3.29	3.26	3.11	3.20
Outlier (%)	0.09	0.09	0.09	0.09	0.09	0.09	0.16	0.09	0.12	0.09

Supplementary Table 4: Refinement statistics for SARS-CoV-2 Spike protein at 37°C.
