# Supplementary material for: Real-space heterogeneous reconstruction, refinement, and disentanglement of CryoEM conformational states with HetSIREN
Source: Nat Commun. 2025 Apr 22;16:3751. doi: 10.1038/s41467-025-59135-0 (PMC12015509; doi:10.1038/s41467-025-59135-0)
Supplement: Supplementary file 2 — Reporting Summary [file 41467_2025_59135_MOESM2_ESM.pdf]

nature portfolio

Corresponding author(s): David Herreros

Last updated by author(s): Mar 6, 2025

Reporting Summary

Nature Portfolio wishes to improve the reproducibility of the work that we publish. This form provides structure for consistency and transparency in reporting. For further information on Nature Portfolio policies, see our Editorial Policies and the Editorial Policy Checklist.

Statistics

For all statistical analyses, confirm that the following items are present in the figure legend, table legend, main text, or Methods section.

n/a	Confirmed	
☐	☒	The exact sample size (*n*) for each experimental group/condition, given as a discrete number and unit of measurement
☒	☐	A statement on whether measurements were taken from distinct samples or whether the same sample was measured repeatedly
☐	☒	The statistical test(s) used AND whether they are one- or two-sided<br>*Only common tests should be described solely by name; describe more complex techniques in the Methods section.*
☒	☐	A description of all covariates tested
☒	☐	A description of any assumptions or corrections, such as tests of normality and adjustment for multiple comparisons
☐	☒	A full description of the statistical parameters including central tendency (e.g. means) or other basic estimates (e.g. regression coefficient) AND variation (e.g. standard deviation) or associated estimates of uncertainty (e.g. confidence intervals)
☐	☒	For null hypothesis testing, the test statistic (e.g. *F*, *t*, *r*) with confidence intervals, effect sizes, degrees of freedom and *P* value noted<br>*Give P values as exact values whenever suitable.*
☒	☐	For Bayesian analysis, information on the choice of priors and Markov chain Monte Carlo settings
☒	☐	For hierarchical and complex designs, identification of the appropriate level for tests and full reporting of outcomes
☒	☐	Estimates of effect sizes (e.g. Cohen's *d*, Pearson's *r*), indicating how they were calculated

Our web collection on statistics for biologists contains articles on many of the points above.

Software and code

Policy information about availability of computer code

Data collection	- GR:Hsp90:FKBP51 data was collected at UCSF as described in CM Noddings, et. al. CryoEM reveals how Hsp90 and FKBP immunophilins co-regulate the glucocorticoid receptor. Nature Structural & Molecular Biology, 30:1867–1877, 2023.<br><br>- SARS-CoV-2 data was collected at Institute of Biological Chemistry, Academia Sinica as described in the section "Cryo-EM sample preparation and data collection for the SARS-CoV-2 samples" from the submitted manuscript
Data analysis	All the data was analyzed with Scipion 3.8.0 software package. Inside Scipion, CryoSPARC 4.5.1, Relion 4.0, and Xmipp 3.24.12.0 packages were also used to process the data.

For manuscripts utilizing custom algorithms or software that are central to the research but not yet described in published literature, software must be made available to editors and reviewers. We strongly encourage code deposition in a community repository (e.g. GitHub). See the Nature Portfolio guidelines for submitting code & software for further information.

Data

Policy information about availability of data

All manuscripts must include a data availability statement. This statement should provide the following information, where applicable:

- Accession codes, unique identifiers, or web links for publicly available datasets
- A description of any restrictions on data availability
- For clinical datasets or third party data, please ensure that the statement adheres to our policy

The atomic coordinates and cryo-EM density maps for the SARS-CoV-2 Spike protein at 4°C and 37°C were deposited in the Protein Data Bank and EM Data Bank with codes 9GDX [https://doi.org/10.2210/pdb9GDX/pdb] and 9GDY [https://doi.org/10.2210/pdb9GDY/pdb] and EMD-51279 [https://www.ebi.ac.uk/emdb/EMD-51279] and EMD-51280 [https://www.ebi.ac.uk/emdb/EMD-51280], respectively.

The synthetic and ribosome dataset analyzed in this work can be downloaded as a Scipion test dataset through the following command: scipion3 testdata --download FlexHub_Tutorials (assuming Scipion is already installed in the system). The atomic model used in the synthetic dataset is deposited in the Protein Data Bank with code 4AKE [https://doi.org/10.2210/pdb4AKE/pdb].

The source data underlying Figures 4b, 6b, 10b and Supplementary Figure 7a, 7b, 7d are provided as a Source Data file.

Research involving human participants, their data, or biological material

Policy information about studies with human participants or human data. See also policy information about sex, gender (identity/presentation), and sexual orientation and race, ethnicity and racism.

Reporting on sex and gender	Not applicable
Reporting on race, ethnicity, or other socially relevant groupings	Not applicable
Population characteristics	Not applicable
Recruitment	Not applicable
Ethics oversight	Not applicable

Note that full information on the approval of the study protocol must also be provided in the manuscript.

Field-specific reporting

Please select the one below that is the best fit for your research. If you are not sure, read the appropriate sections before making your selection.

☒ Life sciences ☐ Behavioural & social sciences ☐ Ecological, evolutionary & environmental sciences

For a reference copy of the document with all sections, see nature.com/documents/nr-reporting-summary-flat.pdf

Life sciences study design

All studies must disclose on these points even when the disclosure is negative.

Sample size	This information is not relevant for image processing data.
Data exclusions	No data was excluded from the analyses.
Replication	All experiments were repeated more than once to ensure that the results and conclusions derived from them were reproducible.
Randomization	This is not relevant to this study, as the datasets are independent and cannot be mixed into groups.
Blinding	This is not relevant for this study, as the datasets are not blind during their analysis.

Reporting for specific materials, systems and methods

We require information from authors about some types of materials, experimental systems and methods used in many studies. Here, indicate whether each material, system or method listed is relevant to your study. If you are not sure if a list item applies to your research, read the appropriate section before selecting a response.

Materials & experimental systems

n/a	Involved in the study
☒	☐ Antibodies
☒	☐ Eukaryotic cell lines
☒	☐ Palaeontology and archaeology
☒	☐ Animals and other organisms
☒	☐ Clinical data
☒	☐ Dual use research of concern
☐	☐ Plants

Methods

n/a	Involved in the study
☒	☐ ChIP-seq
☒	☐ Flow cytometry
☒	☐ MRI-based neuroimaging

Plants

Seed stocks	Not applicable
Novel plant genotypes	Not applicable
Authentication	Not applicable
